# Biofabrication of Prevascularised Hypertrophic Cartilage Microtissues for Bone Tissue Engineering

**DOI:** 10.3389/fbioe.2021.661989

**Published:** 2021-06-07

**Authors:** Jessica Nulty, Ross Burdis, Daniel J. Kelly

**Affiliations:** ^1^Trinity Centre for Biomedical Engineering, Trinity Biomedical Sciences Institute, Trinity College Dublin, Dublin, Ireland; ^2^Department of Mechanical, Manufacturing and Biomedical Engineering, School of Engineering, Trinity College Dublin, Dublin, Ireland; ^3^Advanced Materials and Bioengineering Research Centre (AMBER), Royal College of Surgeons in Ireland and Trinity College Dublin, Dublin, Ireland; ^4^Department of Anatomy and Regenerative Medicine, Royal College of Surgeons in Ireland, Dublin, Ireland

**Keywords:** prevascularisation, microtissues, developmental engineering, bone tissue engineering, biofabrication, bioprinting

## Abstract

Bone tissue engineering (TE) has the potential to transform the treatment of challenging musculoskeletal pathologies. To date, clinical translation of many traditional TE strategies has been impaired by poor vascularisation of the implant. Addressing such challenges has motivated research into developmentally inspired TE strategies, whereby implants mimicking earlier stages of a tissue’s development are engineered *in vitro* and then implanted *in vivo* to fully mature into the adult tissue. The goal of this study was to engineer *in vitro* tissues mimicking the immediate developmental precursor to long bones, specifically a vascularised hypertrophic cartilage template, and to then assess the capacity of such a construct to support endochondral bone formation *in vivo*. To this end, we first developed a method for the generation of large numbers of hypertrophic cartilage microtissues using a microwell system, and encapsulated these microtissues into a fibrin-based hydrogel capable of supporting vasculogenesis by human umbilical vein endothelial cells (HUVECs). The microwells supported the formation of bone marrow derived stem/stromal cell (BMSC) aggregates and their differentiation toward a hypertrophic cartilage phenotype over 5 weeks of cultivation, as evident by the development of a matrix rich in sulphated glycosaminoglycan (sGAG), collagen types I, II, and X, and calcium. Prevascularisation of these microtissues, undertaken *in vitro* 1 week prior to implantation, enhanced their capacity to mineralise, with significantly higher levels of mineralised tissue observed within such implants after 4 weeks *in vivo* within an ectopic murine model for bone formation. It is also possible to integrate such microtissues into 3D bioprinting systems, thereby enabling the bioprinting of scaled-up, patient-specific prevascularised implants. Taken together, these results demonstrate the development of an effective strategy for prevascularising a tissue engineered construct comprised of multiple individual microtissue “building blocks,” which could potentially be used in the treatment of challenging bone defects.

## Introduction

Tissue engineering has the potential to transform the treatment of challenging musculoskeletal pathologies, such as large bone defects and non-union fractures. To date, substantial progress has been made in the field of bone tissue engineering, particularly in identifying suitable cell sources (Caplan, 1991; Li et al., 2007; Kahle et al., 2010; Csobonyeiova et al., 2017) and growth factors (Zhang et al., 2019), as well as developing osteoinductive biomaterials to promote bone formation (Yamasaki and Sakai, 1992; Deng et al., 2017; Walsh et al., 2019). Despite these many advances, there are limited examples of cell-based bone tissue engineering strategies successfully translating into the clinic (Evans, 2011; Woodruff et al., 2012; Mishra et al., 2016; Mittwede et al., 2018). The reasons for this are multi-faceted, but include difficulties in engineering *in vitro* tissues of the scale necessary to treat large bone defects, and ensuring sufficient blood supply to the construct upon implantation into the body (Almubarak et al., 2016; Bienert, 2019). Therefore, there is an urgent need for novel approaches to engineer vascularised tissues of the scale necessary to treat critically sized defects.

3D cell spheroids and aggregates have been utilised by cell biologists and tissue engineers over the last 20 years to study cell-cell interactions, cell differentiation, and as a model system for drug screening (Johnstone et al., 1998; Korff and Augustin, 1998; Friedrich et al., 2009; Huang et al., 2012; Walser et al., 2013; Wenzel et al., 2014). In recent years, there has been an increased interest in using similar spheroidal systems as the building blocks for organogenesis (Mironov et al., 2009; Burdis and Kelly, 2020). This concept leverages the capacity of cells to self-assemble and produce cellular spheroids/aggregates, which are then stimulated to generate microtissues or organoids *in vitro*. These modular units can be combined and, *via* self-organisation, fuse to form a larger engineered tissue capable of continuing along a defined pathway *in vivo*. Multiple methods have been described in the literature to produce multicellular spheroids (Lin and Chang, 2008), including hanging drop (Yoon et al., 2012), spinner culture (Nyberg et al., 2005), and micromoulds (Lopa et al., 2015), enabling the development of tissue engineered grafts for multiple tissues such as cartilage (Huang et al., 2013), skin (Furukawa et al., 2001), myocardium (Chimenti et al., 2011), and bone (Nilsson Hall et al., 2020). There are many advantages to using spheroids over traditional tissue engineering approaches, which typically use a population of (physically separated) cells suspended in biomaterials. Firstly, in the absence of a substrate biomaterial, cells are forced to interact with one another. This process mimics the cellular aggregation that occurs during embryonic development and drives self-organisation (Athanasiou et al., 2013; Laschke and Menger, 2017). It has been shown that the differentiation potential of mesenchymal/marrow stem/stromal cells (MSCs) increases during spheroidal culturing compared to 2D monolayer culture (Kapur et al., 2012; Yoon et al., 2012; Zhang S. et al., 2015). Moreover, using microtissues as “building blocks” has been demonstrated as a viable means of creating large, engineered tissues at therapeutic scales (Skylar-Scott et al., 2019). However, as such constructs increase in size, the overall nutritional demands of the engineered tissue will increase, necessitating novel approaches to vascularise such constructs prior to their implantation into the body. This is particularly true when using cellular aggregates and spheroids for the treatment of large bone defects, which reach many centimetres in length and therefore represent a particularly challenging environment for implanted tissues to remain viable and execute their intended function. This motivates the development of biofabrication strategies to prevascularise engineered grafts consisting of cellular aggregates/spheroids primed for bone formation.

Multiple strategies have been proposed to address the challenge of vascularisation in tissue engineering, from the incorporation of potent angiogenic growth factors, to the inclusion of hollow channels within engineered constructs to stimulate endogenous host angiogenic responses (Murphy et al., 2004; Kempen et al., 2009; Zhang et al., 2014; Zhang W. et al., 2015; Feng et al., 2017; Daly et al., 2018). However, these approaches rely on the growth of host vessels from the periphery of the defect site into a biomaterial or tissue engineered construct. This ingrowth of native vasculature is relatively slow. As such, reliance on host vascularisation often fails to meet the nutrient and oxygen demands of implanted tissues and/or the endogenous reparative cells that migrate into the defect site. Furthermore, the use of exogenous growth factors carries the significant risk of off-target effects and may be unsuitable in cases such as bone resection due to osteosarcomas (Carragee et al., 2011; Weiss, 2015). To address these shortcomings, different strategies have been proposed to “prevascularise” a tissue engineered construct. Their aim is to establish a microvasculature within a construct which can undergo anastomosis with host vessels at the periphery of the construct, leading to a more rapidly perfused engineered tissue (Laschke et al., 2011; Liu et al., 2017). One obstacle in the field for producing prevascularised bone implants is optimising the culturing regime. The induction factors traditionally used to engineer bone tissue and enhance vascular growth can be mutually inhibitory when combined (Correia et al., 2011). Studies investigating culture regimes to engineer vascularised bone *in vitro* have demonstrated that to obtain the concurrent formation of bone and vascular compartments in the same scaffold, osteogenesis and vasculogenesis require sequential induction using appropriate cues (Correia et al., 2014, 2011). Although emerging strategies are investigating the use of other exogenous growth factors which may simultaneously promote osteogenic differentiation whilst supporting vascularisation, such as platelet-derived growth factor (PDGF) (Hutton et al., 2013), there is still a need for strategies that can fabricate vascularised bone tissue of scale.

**FIGURE 1:**
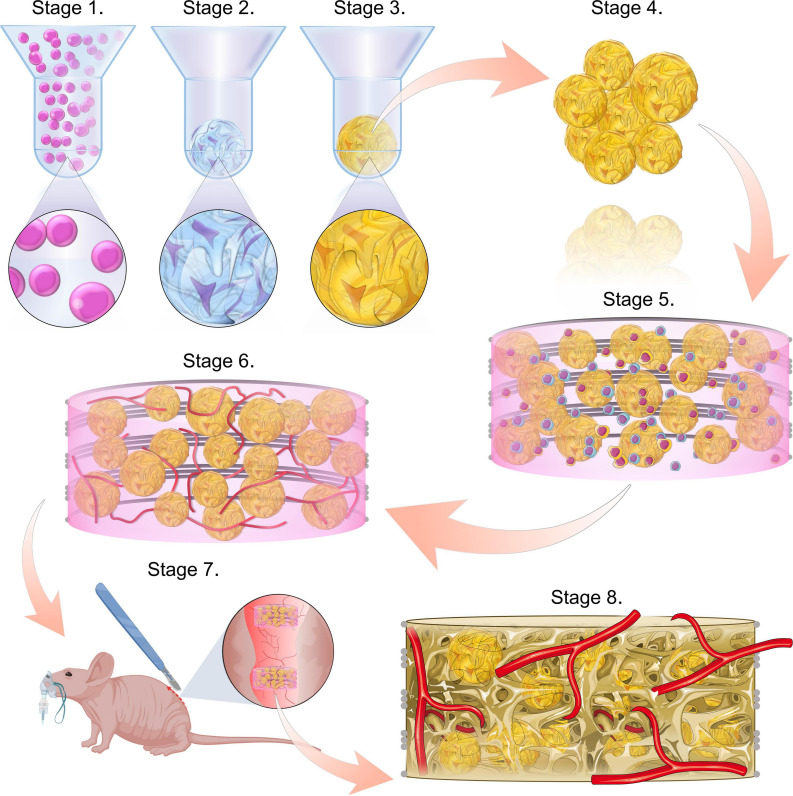
Study schematic. Stages 1–3 consist of the formation of the microtissues, starting with cell seeding the microwells (stage 1), followed by the chondrogenic differentiation of the cell spheroids (stage 2) and ultimately the hypertrophic maturation of the cartilage microtissues (stage 3). Stage 4 involves harvesting the hypertrophic cartilage microtissues from the microwells. These are then combined with a co-culture of HUVECs and hMSCs within a bioink and incorporated into a PCL framework (stage 5). This implant is then cultured for a further week, in the presence of VEGF to form a prevascular network (stage 6). This prevascularised implant is then ready for implantation (stage 7). Bone formation and vascularisation within the implants are evaluated at 4- and 8-week time points (stage 8).

The overall objective of this study was to develop a strategy to prevascularise tissue engineered constructs that were fabricated by combining multiple microtissues into a single implant. With the eventual goal of treating large bone defects, our specific aim was to prevascularise constructs consisting of multiple hypertrophic cartilage microtissues, each capable of bone formation *via* endochondral ossification (Scotti et al., 2013; Sheehy et al., 2015a; Thompson et al., 2015; Daly et al., 2018; Nilsson Hall et al., 2020; Figure 1). As such, we sought to mimic normal long bone development, whereby vascular invasion into a cartilage anlage triggers calcification and endochondral ossification (Mackie et al., 2008; Kronenberg, 2003). To this end, hypertrophic cartilage microtissues were generated *in vitro* using a non-adhesive micro-moulding technique and were then embedded within a fibrin-gelatin-hyaluronic acid hydrogel containing HUVECs and MSCs. This implant was then cultured for 7 days in the presence of VEGF, which induces the formation of a microvascular network. These constructs were then implanted into a subcutaneous mouse model to investigate whether this approach can lead to accelerated vascularisation and mineralisation of the engineered construct *in vivo*.

## Materials and Methods

### Isolation and Expansion of BMSCs

Goat BMSCs (gBMSCs) were harvested under sterile conditions from the sternum of skeletally mature, female, Saanen goats. Briefly, excised bone marrow was cut into small pieces using a scalpel. The marrow pieces were then gently rotated for 5 min in high glucose Dulbecco’s modified eagle’s medium (hgDMEM) GlutaMAX supplemented with 10% v/v FBS, 100 U mL^–1^ penicillin, 100 μg mL^–1^ streptomycin (all Gibco, Biosciences, Dublin, Ireland) and 5 ng mL^–1^ bFGF2 (Prospect Bio) (XPAN) to help liberate the cellular components. The culture medium was then aspirated and passed through a 40 μm cell sieve prior to counting and plating at a density of 57 × 10^3^/cm^2^ and expanded under physioxic conditions (37°C in a humidified atmosphere with 5% CO_2_ and 5 % pO_2_) for improved chondrogenic differentiation. Following colony formation, gBMSCs were trypsinised using 0.25% (w/v) Trypsin Ethylenediaminetetraacetic acid (EDTA). gBMSCs for microtissues were expanded from an initial density of 5,000 cells/cm^2^ in XPAN medium under physioxic conditions until P3. gBMSCs were initially used to validate the capacity of the microwells to support initial cell-aggregation and subsequent growth under chondrogenic culture conditions, while human BMSCs (hBMSCs; see below) were used for all other studies.

hBMSCs were isolated from unprocessed human bone marrow aspirate (Lonza) on the basis of plastic adherence. Briefly, unprocessed bone marrow was plated at 2.5 × 10^5^ cells/cm^2^ (estimated ~4,000–5,000 MSCs/cm^2^) in XPAN medium and expanded under physiological oxygen conditions (37°C in a humidified atmosphere with 5% CO_2_ and 5% pO_2_). Following colony formation, hBMSCs were trypsinised using 0.25% (w/v) Trypsin Ethylenediaminetetraacetic acid (EDTA) and tripotentiality was confirmed as previously described (Vinardell et al., 2009). hBMSCs used within the fibrin-based hydrogel to help support the development of a vascular network *in vitro* (see below) were seeded at 5,000 cells/cm^2^ XPAN and expanded under normoxic conditions (37°C in a humidified atmosphere with 5% CO_2_ and 20% pO_2_) and used at P4. hBMSCs used for hypertrophic cartilage spheroids were expanded in the same media but under lower oxygen conditions (5% pO_2_) and aggregated into microtissues at P3.

### Endothelial Expansion

Human Umbilical Vein Endothelial cells (HUVECs) (Lonza, Walkersville, MD) were cultured at 2,500 cells/cm^2^ in Endothelial Growth Medium (EGM-2) which had been supplemented with EGM-2 BulletKit (Lonza) at 37°C in a humidified atmosphere with 5 % CO_2_ and 20 % pO_2_. HUVECs were used at P4.

### Hypertrophic Cartilage Microtissue Production

#### Moulding Assembly Design and Fabrication

Positive moulds were first designed using Solidworks CAD software. Briefly, moulds were designed as a stamp intended to imprint molten agarose with the desired microwell design. Each of the 401 microwells measured 1 mm in diameter (ø), had a total well depth of 1.5 mm—comprised of a 1 mm deep cylindrical section and a domed end which was 0.5 mm deep. The top of each microwell was a chamfered asymmetrically, 0.5 mm × 1 mm (width × depth), ensuring there were no flat sections of the mould between adjacent microwells. Positive moulds were fabricated using a Form 2 stereolithography (SLA) printer (Formlabs, Massachusetts, United States). Prior to printing, a STL file for the part was prepared using Preform 2.16.0 software (Formlabs, Massachusetts, United States), setting a 50 μm layer height defined the resolution of the print. Completed parts were processed post-printing in accordance with the manufacturer’s guidelines. Briefly, parts were washed in propan-2-ol (Sigma Aldrich) to clear any uncured resin, following which they were exposed to UV light (405 nm, 9.1 W) (Form cure, Formlabs, Massachusetts, United States) for 30 min at 60°C to ensure complete crosslinking. Moulds were cleaned, and gas sterilised using ethylene oxide (EtO) prior to use (Anprolene^®^ gas sterilisation cabinet, Andersen Sterilizers).

#### Non-adherent Hydrogel Microwells

**FIGURE 2:**
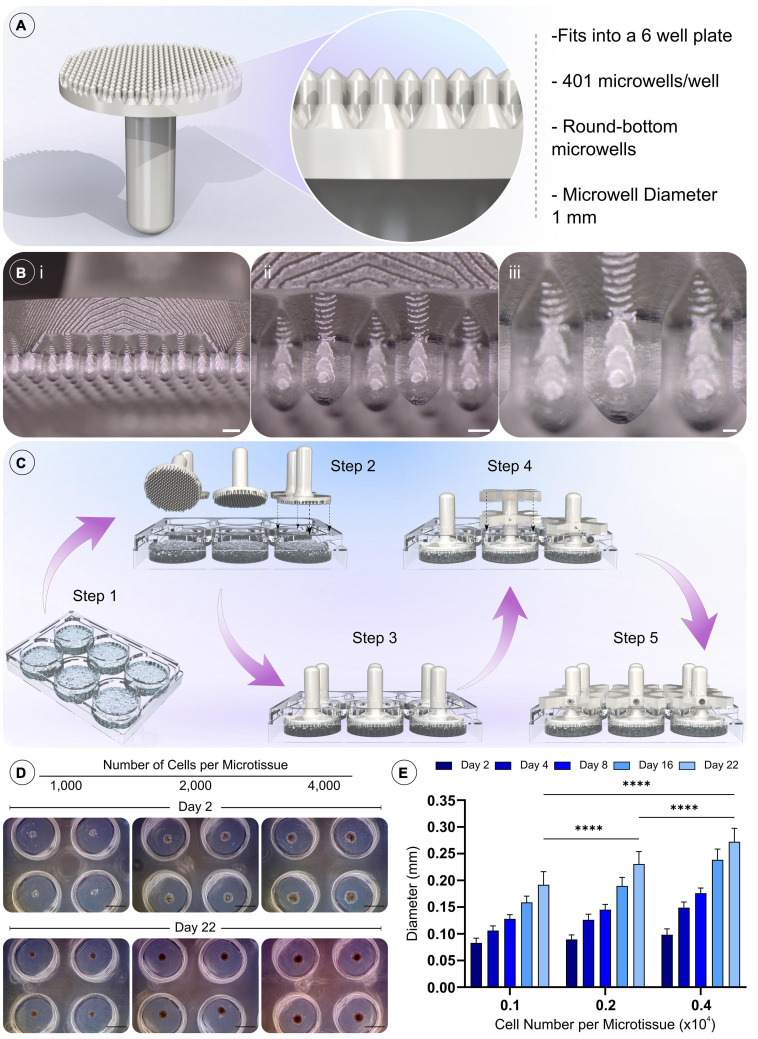
Design of novel microwell moulds, the methodology for fabricating non-adherent microwells, and the validation of cell spheroid formation. A) Model and details of the positive mould used to generate the microwells. **(Bi–iii)** Macroscopic images of the 3D printed positive mould (Scale bar = 1 mm, 500 μm, and 200 μm, respectively). **(C)** Procedure for moulding the microwells. Step 1 molten agarose is added to the wells of a 6 well plate, then positive moulds are then pressed into the still molten agarose (steps 2–3). Holders are placed over the positive moulds and a grub screw inserted into the holder to prevent subsidence of the positive mould as the agarose cools (step 4). The final assembly is shown in step 5, the positive moulds are left in place until the agarose cools and solidifies. Upon removal, the cast agarose within the well is patterned with the microwell design. **(D)** Validation of the formation of gBMSC spheroids after 48 h, and their growth over 21 days of chondrogenic culture (Scale bar = 500 μm). **(E)** Quantification of spheroid diameter change, ^****^denotes significance two-way ANOVA with Tukey *post hoc* test, *p* < 0.0001 (*n* = 25, mean ± SD).

Agarose microwells were fabricated under sterile conditions, within a class II biosafety cabinet. Firstly, agarose (Sigma-Aldrich) was dissolved in phosphate buffered saline (PBS) (Sigma-Aldrich) at a concentration of 4% (w/v) and autoclaved to ensure sterility. 6 mL of molten agarose solution was pipetted into a well of a 6 well plate (Nunc 6-Well Plate, Round, Thermo Fisher Scientific, Massachusetts, United States) (Figure 2). The aforementioned sterile 3D printed positive moulds were then inserted into the agarose, ensuring no bubbles became trapped underneath the mould. A holder was then placed over mould and a M2 screw tightened to secure the height and position of the mould. This process was repeated for each well, ensuring the height of the moulds are equal. Once cooled, excess solidified agarose above the mould was removed and the positive mould was pulled from the agarose, leaving the patterned agarose imprinted with the microwells within each well. All agarose microwells were soaked overnight in DMEM prior to cell seeding.

#### Cell Seeding and Hypertrophic Cartilage Priming

Cells were seeded into the microwells by pipetting an appropriate density (1 × 10^3^ cells mL^–1^, 2 × 10^3^ cells mL^–1^ or 4 × 10^3^ cells/microwell) into each well. After seeding, plates were centrifuged at 700 × g for 5 min to collect cells at the bottom of each well. Plates were then incubated in XPAN described in §3.1 overnight to allow condensation to occur (Supplementary Figure 2) before switching to chondrogenic media consisting of hgDMEM GlutaMAX supplemented with 100 U mL^–1^ penicillin, 100 μg mL^–1^ streptomycin (both Gibco), 100 μg mL^–1^ sodium pyruvate, 40 μg mL^–1^ L-proline, 50 μg mL^–1^ L-ascorbic acid-2-phosphate, 4.7 μg mL^–1^ linoleic acid, 1.5 mg mL^–1^ bovine serum albumin, 1 × insulin–transferrin–selenium, 100 nM dexamethasone (all from Sigma), 2.5 μg mL^–1^ amphotericin B and 10 ng mL^–1^ of human transforming growth factor-β3 (TGF-β3) (Peprotech, United Kingdom) at 37°C in a humidified atmosphere with 5 % CO_2_ and 5 % pO_2_ for 3 weeks. Microtissues were then moved to 20 % pO_2_ and switched to hypertrophic media consisting of hgDMEM GlutaMAX supplemented with 100 U mL^–1^ penicillin, 100 μg mL^–1^ streptomycin (both Gibco), 1 × insulin–transferrin–selenium, 4.7 μg mL^–1^ linoleic acid, 50 nM thyroxine, 100 nM dexamethasone, 250 μM ascorbic acid, 7 mM β-glycerophosphate and 2.5 μg mL^–1^ amphotericin B (all from Sigma) for a further 2 weeks.

#### Harvesting Hypertrophic Cartilage Microtissues

After 5 weeks, microtissues were liberated from the microwells by first flushing media over the surface of the agarose using a pipette. The culture media was then removed from each well, and the agarose removed and inverted into a new 6 well plate. This plate was centrifuged at 700 × g for 5 min to collect the microtissues in the bottom of the well plate. Fresh culture media was washed over the surface of the wells of the plate to collect the microtissues. The suspension of microtissues was then passed through an appropriately sized pluriStrainer^®^ (pluriSelect^®^, Leipzig, Germany) to capture any agarose fragments. The density of this purified microtissue suspension could then be quantified and combined with the fibrin-based hydrogel to generate prevascularised implants and implants containing microtissues only (section “Biofabrication of Prevascularised Constructs”). *In vitro* histology samples were prepared by combining hypertrophic cartilage microtissues with the fibrin-based hydrogel (section Babrication of Fibrin-Gelatin-Hyaluronic Acid Hydrogels and Bioinks), fixing in 4% PFA overnight at 4°C, and evaluating histologically (section “Histological Analysis”).

### Fabrication of Fibrin-Gelatin-Hyaluronic Acid Hydrogels and Bioinks

To enable the use of fibrin as a printable bioink, a gelatin carrier method was adapted from Kang et al. (2016). Briefly, hyaluronic acid (Sigma) was added to high glucose Dulbecco’s Modified Eagle Medium (hgDMEM) (Gibco, Biosciences) at a concentration of 3 mg mL^–1^ and stirred overnight at 37°C. 10% (v/v) glycerol (Sigma) was then added and the solution was stirred for 1 h at room temperature. Gelatin type A (175 g bloom) (Sigma) was added at a concentration of 40 mg mL^–1^ and stirred for 2 h at 37°C until fully dissolved. Before use, Fibrinogen (Sigma) was added to this thawed carrier gel at a concentration of 30 mg mL^–1^ and stirred for 2 h at 37°C. To enhance the angiogenicity of the bioink, D-erythro-sphingosine-1-phosphate (S1P; Avanti Polar lipids, AL, United States) was added at a concentration of 125 μM mL^–1^. For both cast constructs and printed constructs this fibrin-gelatin-hyaluronic acid formulation (from here on in termed a “fibrin-based” hydrogel or bioink) was used. To fabricate the prevascularising (PV) bioink, HUVECs and hBMSCs were loaded into the bioink at 3 × 10^6^ cells mL^–1^ and 1.5 × 10^6^ cells mL^–1^, respectively, as previously described (Nulty et al., 2021).

### Micro-Computed Tomography Scan Conversion to G-Code

A micro-computed tomography (μCT) scan of a rat femur was first segmented and converted to STL file format using 3D Slicer free software^1^. This STL was then converted to G code using BioCAM^TM^ and BioCAD^TM^ software packages (RegenHU, Switzerland). This scan was carried out as part of a protocol and study that were reviewed and approved by the ethics committee of Trinity College Dublin and the Health Products Regulatory Authority (HPRA) in Ireland (Approval—AE19136/P087).

### Biofabrication of Prevascularised Constructs

Microtissue constructs were fabricated by both casting and by 3D bioprinting. Hypertrophic microtissues were loaded into either a fibrin-based hydrogel/bioink (microtissue only group) or a PV hydrogel/bioink (PV microtissue group) at 17 × 10^3^ spheroids mL^–1^ (680/Construct). For the PV only group, PV bioink was used without any additional spheroids. Both cast and bioprinted constructs were supported by a 3D printed polycaprolactone (PCL) scaffold. Briefly, scaffolds were designed using BioCAD^TM^ software package and produced using the 3D Discovery multi-head bioprinting system (Regen Hu, Switzerland). Porous PCL (CAPA Ingevity, SC, United States) disc scaffolds were printed at 80°C at 0.5 MPa using a 27G needle (1 mm height, 4 mm diameter). Scaffolds were sterilised using ethylene oxide (EtO) gas prior to the addition of cell-laden hydrogel/bioinks. For cast constructs, a volume of 12.6 μL of cell/spheroid-laden hydrogel was manually deposited to fill the cylindrical PCL scaffolds. For bioprinted constructs, the fibrin-based bioinks were printed using a pneumatic driven syringe with a 25G needle at temperatures between (10–30)°C under pressures of between 0.05 and 0.2 MPa. To assist the accuracy of the printing process, 4 % (w/v) agarose (Sigma) well inserts were fabricated using custom-designed moulds printed with Form2 SLA printer (FormLabs, MA, United States). These agarose inserts were soaked in thrombin (20 U mL^–1^) prior to printing, as a means of improving print fidelity of the fibrin-based bioink. After printing/casting, all constructs were immersed in a thrombin bath (20 U mL^–1^) for 30 min at RT to allow the thrombin-catalysed polymerisation of fibrinogen to fibrin. The 3D Discovery was encased in a laminar flow hood to ensure sterility throughout the biofabrication process.

### *In vivo* Study Design

To investigate the effect of prevascularisation on a cartilage template *in vivo*, three groups were compared: A prevascularised fibrin-based hydrogel disc (PV only); a fibrin-based hydrogel disc containing hypertrophic microtissues (microtissues only); and a prevascularised fibrin-based hydrogel disc containing hypertrophic microtissues (PV microtissues). All disc constructs were fabricated by casting the microtissue laden hydrogels into moulds as described in section “Biofabrication of Prevascularised Constructs”. In addition, 3D bioprinting (see section “Biofabrication of Prevascularised Constructs”) was used to generate constructs mimicking the geometry of a segment of a rat femur. All constructs were then cultured in EGM-2 media supplemented with 50 ng mL^–1^ of VEGF for 7 days under normoxic conditions to allow for the formation of microvessels. Constructs were then implanted subcutaneously into nude mice (see section *In vivo* Subcutaneous Implantation) and explanted at 4 and 8 weeks for analyses.

### *In vivo* Subcutaneous Implantation

Constructs were implanted subcutaneously into the back of Balb/c female nude mice (Harlan, United Kingdom). Briefly, two subcutaneous pockets were made along the central line of the spine, one at the shoulders and the other at the hips. Two constructs were inserted into each pocket. Six constructs were implanted per group and constructs were harvested 2, 4, and 8 weeks post-implantation. Mice were anaesthetised using an intraperitoneal injection of xylazine hydrochloride and ketamine hydrochloride, Carprofen was added to water for 24 h post-surgery, and mice were sacrificed by CO_2_ inhalation. This protocol and study were reviewed and approved by the ethics committee of Trinity College Dublin and the Health Products Regulatory Agency (HPRA, approval number AE19136/P069). Post-implantation samples were fixed in 4% PFA for 24 h.

### Micro-Computed Tomography

Micro-computed tomography (μCT) scans were performed using a Scanco Medical 40 μCT system (Scanco Medical, Bassersdorf, Switzerland) in order to visualise and quantify mineral content and to assess mineral distribution within all constructs.

Constructs were scanned in 50% EtOH, at a voxel resolution of 12 μm, a voltage of 70 kVp, a current of 114 μA. Reconstructed 3D images were generated to visualise the mineral content and distribution throughout the constructs. A Gaussian filter (sigma = 1.2, support = 2) was used to suppress noise and a global threshold of 482.

### Histological Analysis

*In vivo* samples were decalcified using “Decalcifying Solution-Lite” (Sigma) for approximately 1 week. Samples were frequently x-rayed to determine if any mineral content remained. When no mineral was visible the sample was considered decalcified. All samples (*in vitro* and decalcified *in vivo*) were then dehydrated in graded series of ethanol solutions (70–100%), cleared in xylene, and embedded in paraffin wax (all Sigma-Aldrich). Sections (5 μm) were rehydrated in graded series and stained with haematoxylin and eosin, 1 % (w/v) alcian blue 8GX in 0.1 M HCL to assess sulphated glycosaminoglycan (sGAG) content with a counter stain of 0.1% (w/v) nuclear fast red to assess cellular distribution, 0.1% (w/v) picrosirius red to assess collagen distribution, 0.2% (w/v) Safranin O to assess sGAG content post-implantation and Goldner’s trichrome (Groat’s iron haematoxylin, Fuchsine, Orange G, Fast Green; all from Sigma) for visualising bone (Mineralised bone = dark green, Fibrous Tissue = Light green, Osteoid = orange/red, erythrocytes = dark red). Immunohistochemistry was performed for collagen type I (col I) (Abcam ab90395 1:400), collagen type II (col II) (Santa Cruz- sc52658 1:400), and collagen type X (col X) (Abcam 49945, 1:200). Slides were then imaged using an Aperio ScanScope slide scanner. Samples stained for picrosirius red were imaged using polarised light microscopy (PLM) to determine collagen fibre orientation. *In vivo* sections were evaluated for vessel infiltration by counting vessels visible across an entire section using Aperia ImageScope and ImageJ software. Bone fraction of sections taken of the entire implant was determined using Goldner’s trichrome staining and MIPAR Image Analysis software (MIPAR Software LLC, United States) using native bone as a positive control for thresholding. It should be noted that PCL is cleared during the tissue processing and leaves empty spaces in constructs as a result.

### Statistical Analysis

Statistical analyses were performed using GraphPad Prism software (GraphPad Software, CA, United States). To analyse significant differences between two groups at one timepoint a standard two-tailed *t*-test was performed. To analyse variance between > 2 groups at one timepoint a one-way analyses of variance (ANOVA) was performed with Tukey *post hoc* test. To analyse variance between > 2 groups at multiple timepoints, two-way ANOVA was used with Tukey *post hoc* test. Numerical and graphical results are displayed as mean ± standard deviation unless stated otherwise. Significance was accepted at a level of *p* < 0.05.

## Results

### Development of a Custom Microwell Design to Support Aggregation of BMSCs

Custom-made agarose microwell inserts were fabricated using a 3D printed positive mould (Figure 2). These moulds were designed to fit into a 6 well plate, creating 401 microwells per well (Figure 2A). The designed positive moulds were successfully fabricated using a consumer SLA printer, with excellent resolution and surface finish (Figure 2B). The conceived moulding process is easily implemented and highly reproducible, allowing the production of reliable microwells within each well from a non-adherent material. In addition, the reusable nature of the positive moulds and other assembly components (holders and screw) make this a cost-effective method for generating microwells. The addition of the chamfer to the top of each microwell created an upper surface without flat regions. This in-turn increased the seeding efficiency of the system by collecting all cells into the microwells and preventing their aggregation outside of the well. An early optimisation of the design comparing a chamfer and non-chamfer (flat top) design can be found in the supporting information, where the chamfer plus, centrifugation was found to enhance the development of cellular spheroids (Supplementary Figure 1).

We next sought to validate that the microwells supported the formation of BMSC spheroids and the growth of these cellular aggregates *in vitro*. Over 21 days of culture in chondrogenic media, the cellular spheroids were found to increase in size for all microwell cell seeding densities. Their final size was found to depend on the initial cell number used to generate the spheroid, with higher seeding densities generating larger spheroids (Figures 2D,E).

### hBMSC Spheroids Can Be Differentiated Into Hypertrophic Cartilage Microtissues

**FIGURE 3:**
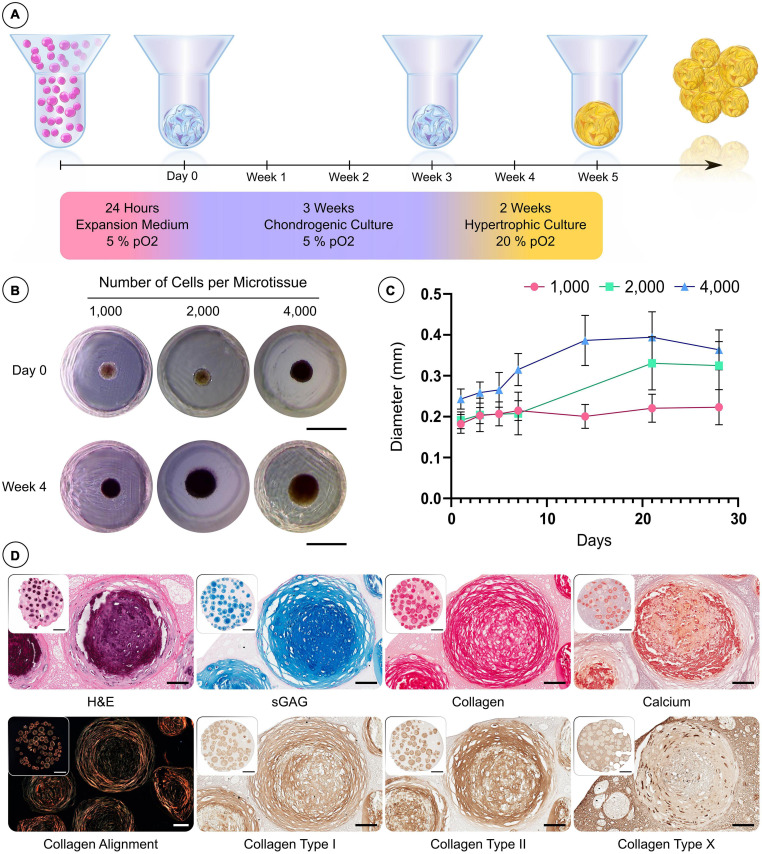
Custom microwell design supports the formation of hBMSC spheroids and their differentiation into hypertrophic cartilage microtissues. **(A)** Schematic of the culture regime employed to generate hypertrophic cartilage microtissue. **(B)** Visual comparison of microtissue diameter of 1,000, 2,000, and 4,000 cells/microtissues at 24 h and 28 days. **(C)** Growth curve of microtissues over 28 days (*n* = 48, Mean ± SD). **(D)** Histological evaluation of microtissues after 5 weeks hypertrophic priming (from left to right—row 1) hematoxylin and eosin (H&E) staining, alcian blue staining, picrosirius red staining and finally alizarin red staining. Row 2—Polarised light microscopy, immunohistochemistry (Scale bar of inset image = 1 mm and high mag image = 100 μm).

We next sought to assess the capacity of the microwell system to support the development of hypertrophic cartilage microtissues using hBMSCs at different cell densities (Figure 3A). The diameters of microtissues containing 1,000, 2,000, and 4,000 cells (per microtissue) were highly uniform after 24 h (SD < 0.025 mm) (Figures 3B,C). All microtissues increased in diameter with time in culture (Figures 3B,C), and following the 5-week culture period, the 1,000, 2,000, and 4,000 cells per microtissue had a mean diameter of 220, 320, and 360 μm, respectively (Figure 3C). To assess tissue development histologically, multiple microtissues were combined within a cell-free fibrin-based hydrogel and processed immediately. The microtissues stained positive for the canonical cartilaginous extracellular matrix markers, sulphated glycosaminoglycan (sGAG) and type II collagen, whilst calcium deposition (Figure 3D) indicated that the *in vitro* culture protocol successfully facilitated the production of hypertrophic cartilage microtissues. The presence of collagen types I and II, and lower levels of collagen type X, was detected using IHC after the 5 weeks of *in vitro* culture (Figure 3D).

### The *in vivo* Mineralisation Potential of Hypertrophic Cartilage Microtissues Is Enhanced by Prevascularisation

**FIGURE 4:**
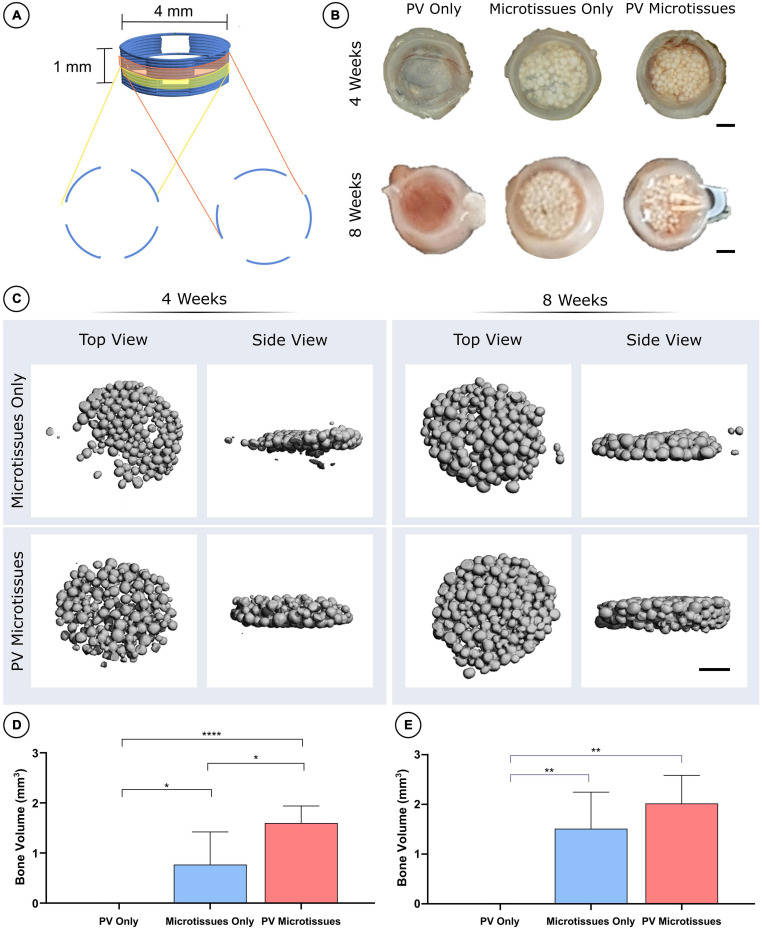
MicroCT analysis of constructs post-implantation. **(A)** Schematic of 3D printed PCL scaffolds. **(B)** Macro images of all three groups at 4 and 8 weeks. Scale bar = 1 mm for all images. **(C)** 3D reconstructions of microCT scans of the microtissues only and PV microtissues groups at 4 and 8 weeks post-implantation. Quantification of bone volume (mm^3^) at **(D)** 4 weeks and **(E)** 8 weeks significant differences are denoted as; *where *p* < 0.05, **where *p* < 0.01, and ****where *p* < 0.0001 when tested using a one-way ANOVA followed by Tukey *post hoc* test (mean ± SD, *n* = 6 for PV microtissues and *n* = 4 for microtissue only and PV only) **(D)**.

Following 5 weeks of *in vitro* priming, microtissues were combined with either a control fibrin-based hydrogel (microtissues only) or a prevascularised fibrin-based hydrogel (PV microtissues; previously described in Nulty et al. (2021) and then cast into 3D printed polycaprolactone (PCL) discs (4 mm (ø) × 1 mm; Figure 4A). The PV fibrin-based hydrogel contained HUVECs and hBMSCs at a concentration of 3 × 10^6^ and 1.5 × 10^6^ cells mL^–1^, respectively. These two groups, along with a PV only control without any microtissues, were cultured for 7 days in EGM-2 media containing VEGF (50 ng mL^–1^) to allow for the formation of a microvascular network. We have previously demonstrated that such a fibrin-based hydrogel can support the development of vascular network when seeded with such a co-culture of HUVECS and BMSCs (Nulty et al., 2021). Micro-computed tomography (μCT) analysis performed on the constructs following 6 weeks of *in vitro* culture showed no detectable mineral deposition (data not shown). All groups were subsequently implanted subcutaneously into nude mice.

Following 4 and 8 weeks *in vivo*, negligible mineral was observed in the PV only controls. In contrast, it could be observed macroscopically that the cartilage microtissues had mineralised *in vivo*, taking on a bony appearance (Figure 4B). μCT analysis confirmed this observation (Figure 4C), with prevascularisation of the microtissues prior to implantation leading to significantly higher levels of mineralisation after 4 weeks *in vivo* (Figures 4C,D). No significant differences in construct mineralisation between the microtissue only group and the prevascularised microtissues group was observed at the 8-week time point. However, there was an increase in bone volume from 4 to 8 weeks in both groups (1.51 ± 0.73 mm^3^ and 1.82 ± 0.54 mm^3^ for microtissues only and PV microtissues, respectively), and a trend toward more bone in the PV microtissue group at the later timepoint (Figures 4C,E).

**FIGURE 5:**
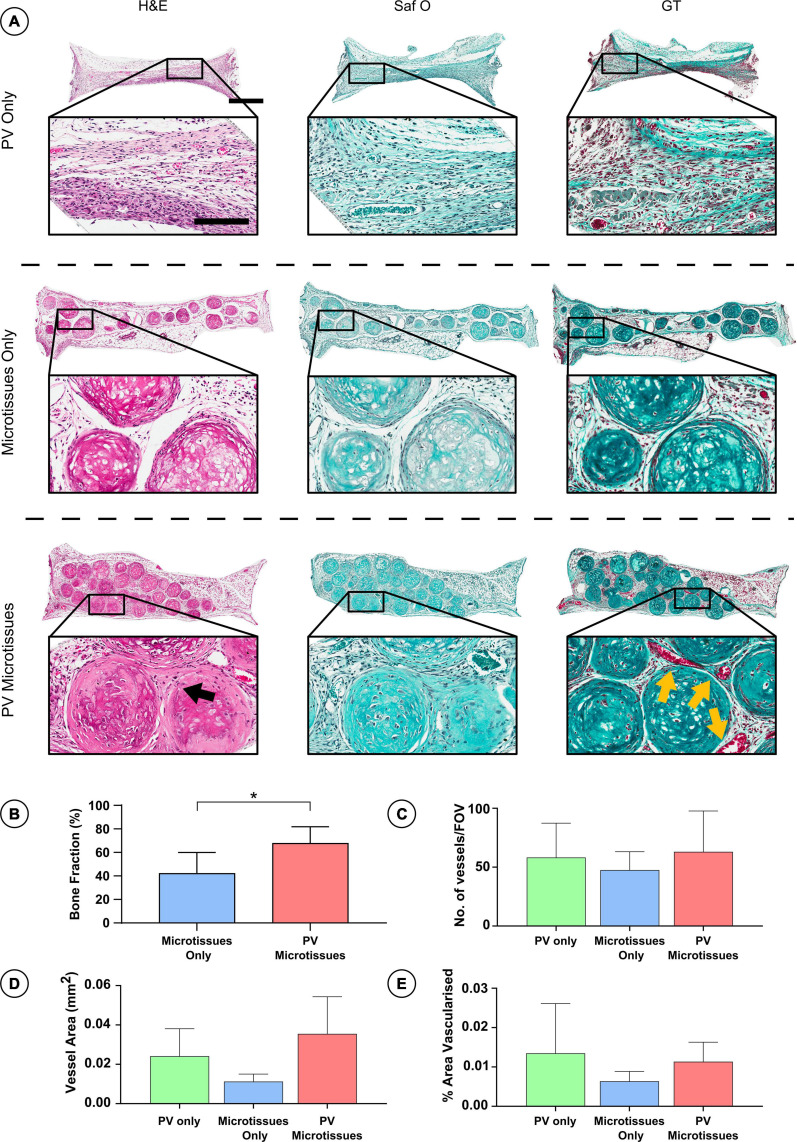
Histological analysis of constructs 4 weeks post-implantation. **(A)** Haematoxylin and eosin staining (H&E), Safranin O staining (Saf O) and Goldner’s Trichome staining (GT) at 4 week Scale bar = 500 μm for low resolution images; scale bar = 150 μm for high resolution images (Black arrows indicate fusion and yellow arrows show blood vessels). **(B)** Quantification of bone fraction **(C)** quantification of vessel no. **(D)** Vessel area and **(E)** percentage vascularised area, *denotes significance two-way ANOVA with Tukey *post hoc* test, *p* < 0.05 (*n* = 6 for PV microtissues and *n* = 4 for microtissue only and PV only, Mean ± SD).

**FIGURE 6:**
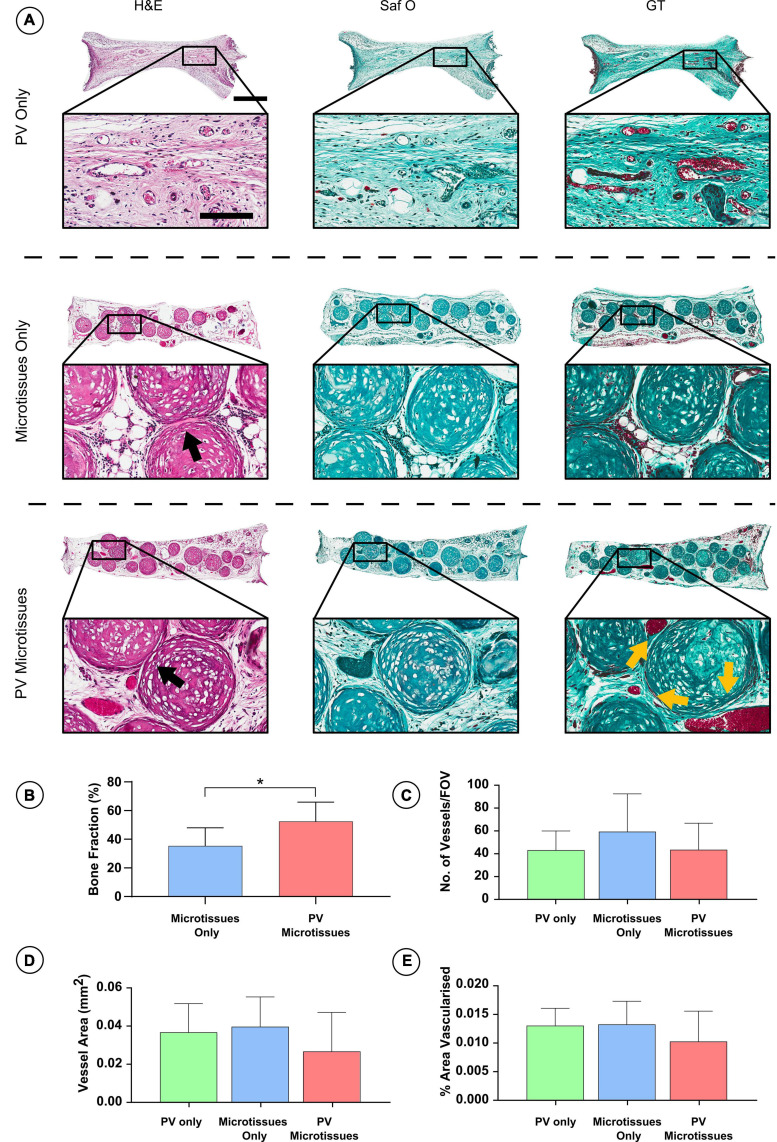
Histological analysis of constructs 8 weeks post-implantation. **(A)** Haematoxylin and eosin staining (H&E), Safranin O staining (Saf O) and Goldner’s Trichome staining (GT) at 8 week Scale bar = 500 μm for low resolution images; scale bar = 150 μm for high resolution images (Black arrows indicate fusion & yellow arrows show blood vessels). **(B)** Quantification of bone fraction **(C)** quantification of vessel no. **(D)** Vessel area and **(E)** percentage vascularised area, *denotes significance two-way ANOVA with Tukey *post hoc* test, *p* < 0.05 (*n* = 6 for PV microtissues and *n* = 4 for microtissue only and PV only, Mean ± SD).

Histological analysis was carried out on the *in vivo* microtissue constructs to investigate vascularisation and bony-tissue formation after subcutaneous implantation. Safranin O staining suggested little or no cartilage remained in any implants (Figures 5A, 6A). Instead, Goldner’s trichome (GT) staining indicated that the hypertrophic microtissues had mineralised by week 4 (Figure 5A), and that this calcified tissue persisted at week 8 (Figure 6A). Blood vessels (yellow arrows) were present in all groups at both time points (Figures 5A, 6A). There was a trend toward increased vascularisation (quantified by a higher number of vessels and larger vessels in the prevascularised groups) at the 4 week time point (Figures 5C–E), although these differences were not statistically significant, and no such trends were observed at the 8 week time point (Figures 6C–E). Fusion between adjacent hypertrophic cartilage spheroids was noted in both microtissue groups at both time points (Black arrows, Figures 5A, 6A). At the earlier *in vivo* time point (4 weeks), histological quantification of bone fraction corroborated the μCT findings, indicating that prevascularisation accelerated mineralisation (Figure 5B). Furthermore, histological analysis at 8 weeks post-implantation demonstrated enhanced mineralisation by the prevascularised cartilage microtissues (Figure 6B).

As well as undertaking this proof of concept study to assess whether prevascularisation enhances the capacity of hypertrophic cartilage microtissues to generate bone *in vivo*, we also assessed whether 3D bioprinting could be leveraged to fabricate larger scale, anatomically defined constructs. To this end, hypertrophic microtissues were bioprinted into a 3D printed polycaprolactone (PCL) scaffold. The geometry of the implant was defined from a scan taken of a healthy rat femoral bone, a region used commonly as a defect model for studying long bone regeneration. Briefly, a μCT scan was taken of a rat femur, this scan was then segmented and converted to STL file format using 3D Slicer software (see text footnote 1). This STL was then converted to G code using BioCAM^TM^ and BioCAD^TM^ software packages and 3D printed (Supplementary Figure 3A). The polymer framework was designed to recapitulate the long bone cortex, as such, the thermopolymer printhead toolpath followed the outer perimeter of the model bone. This created an anatomically accurate PCL shell into which hypertrophic microtissues, combined with a PV bioink containing HUVECs and MSCs, were bioprinted (Supplementary Figure 3B). This bioprinted scaffold was then implanted subcutaneously into a nude mouse. After 4 weeks *in vivo*, there was evidence of mineralisation within construct showing, as proof of principle, that this approach was compatible with 3D printing technology without compromising biological efficacy (Supplementary Figures 3C,D).

## Discussion

Bone tissue engineering aims to overcome the shortcomings and limitations of current treatments for regenerating large bone defects by generating *in vitro* bone graft substitutes. A sufficient supply of nutrients and oxygen is essential for the success of these engineered tissues. This study demonstrates that hypertrophic cartilage microtissues, which can be fabricated *in vitro* and are inherently primed to generate endochondral bone, will calcify upon *in vivo* implantation. The *in vivo* mineralisation potential of these constructs can be further enhanced by prevascularising the microtissues prior to implantation. Additionally, this study shows, as a proof of concept, that this approach can be combined with emerging 3D bioprinting technologies to produce prevascularised tissues mimicking the geometry of a target defect site.

To date, cartilage rudiments for bone tissue engineering have been fabricated *in vitro* using pellets containing large amounts of cells (>2 × 10^5^ cells/pellet) or cells encapsulated within a biomaterial (Scotti et al., 2010, 2013; Bahney et al., 2014; Harada et al., 2014; van der Stok et al., 2014; Cunniffe et al., 2015; Sheehy et al., 2015b). However, the use of large cell numbers can result in inhomogeneous differentiation and matrix deposition, and core cell death due to diffusion-related challenges (Lyons et al., 2010; Bhattacharjee et al., 2015), whilst the use of biomaterials can hinder vascularisation and bone formation *in vivo* due their slow and/or uncontrolled degradation rates (Daly et al., 2018). Here microtissues, with diameters ranging between 220 and 360 μm depending on the initial cell number, were fabricated using hBMSCs and custom-made hydrogel micromoulds. An ideal platform for generating such microtissues has been defined as a scalable process, which can create standardised spheroids with uniform shape, size and fortitude to be used in biofabrication processes such as bioprinting (Mironov et al., 2009). The hydrogel microwell platform developed in this study fit this criteria, offering a simple, yet effective method for generating hypertrophic cartilage microtissues. Specifically, the microwells were designed to generate a standardised and homogenous population of microtissues. To achieve this, key design features such as round-bottom microwells, a sloped entrance to each microwell, sufficient microwell depth, and patterning the entire culture surface were built-in. Other suitable methods for generating large numbers of microtissues for the biofabrication of musculoskeletal tissue have been proposed (De Moor et al., 2018, 2019, 2020). However, these approach involve manual manipulation of the hydrogel microwell mould, whereby the hydrogel microwells are cast in a separate positive mould before being transferred into the well-plate. As such, the direct-moulding approach developed in this work significantly streamlines the process and mitigates human error during sterile fabrication. Despite concerns that the generation of microtissues above 150 μm will also succumb to the nutrient limitations associated with larger engineered tissues and micromass approaches (Laschke and Menger, 2017; Nilsson Hall et al., 2020), we observed no such diffusion-related challenges, with homogenous deposition of hypertrophic cartilage matrix within the microtissues.

The use of a “bulking” biomaterial has been proposed as a means of improving the scalability of microtissue approaches toward multi-centimetre tissue engineering applications (Nilsson Hall et al., 2020). Here, others have successfully leveraged bioprinting to deposit spheroids within a photocurable bioink for cartilage (De Moor et al., 2020) and vascular applications (Benmeridja et al., 2020). In this study, we demonstrate that an *active* cell-laden hydrogel bioink can act as a vehicle for TE and bioprinting applications using microtissues, whilst additionally acting as a substrate to support the development of a vascular network throughout the construct. The numerous (>600) microtissues embedded within such a construct were able to proceed along an endochondral pathway *in vivo* within such a construct. However, evidence of complete fusion between all microtissues following subcutaneous implantation was limited. This may be due to a lack of direct contact between the microtissues within the hydrogel, and fine-tuning the density of the microtissues within the bioink to achieve a more homogenous bony tissue is required. Moreover, it has been shown that the ability of cell aggregates to fuse to one another can diminish over time (Bhumiratana et al., 2014). Decreasing the length of the hypertrophic culture regime may improve the microtissue’s ability to fuse *in vivo*, as the peripheral cells would be less embedded within a dense calcified cartilage matrix. Additionally, implantation of microtissue constructs into a relevant orthotopic location might also lead to greater remodelling of the microtissues *in vivo* and better support homogenous bone development, due to the presence of relevant cell types and signalling factors at the recipient site.

During normal bone development, the process of hypertrophic cartilage turnover and replacement by bone is accompanied by vascular invasion (Kronenberg, 2003). In this study, prevascularising hypertrophic microtissues prior to implantation accelerated mineralisation compared to implantation of microtissues alone. This result is consistent with previous studies that have attempted to prevascularise bone tissue engineering constructs prior to implantation. For example, prevascularising a calcium phosphate cement scaffold with HUVECs and human-induced pluripotent stem cell-derived MSCs has been shown to promote vascularisation and mineralisation in a rat cranial defect model (Liu et al., 2017). The accelerated mineralisation we observed *in vivo* did not correlate with significantly higher levels of implant vascularisation. It may be that prevascularising the cartilage microtissues *in vitro* further primed them for endochondral ossification, leading to accelerated mineralisation of the constructs in the absence of higher levels of vascularisation *in vivo*. Previous studies have demonstrated that HUVECs enhance the activation of endogenous Wnt and BMP signalling and increased ALP expression in hBMSCs (Saleh et al., 2011). It has also been shown that the addition of HUVECs to a cartilage pellet enhances the mineralisation potential of MSCs compared to chondrogenic priming alone (Freeman et al., 2015).

As the hypertrophic chondrocytes that reside within the microtissues are programmed to release angiogenic factors that support vascularisation, it is perhaps unsurprising that prevascularisation of the microtissues did not lead to dramatically higher levels of vascularisation *in vivo*. During the later stages of endochondral ossification, hypertrophic chondrocytes express Osterix, which is a potent inducer of VEGF expression (Tang et al., 2012). This leads to high levels of VEGF within the hypertrophic cartilage which stimulate vessel infiltration (Carlevaro et al., 2000). It should be noted that there was a non-significant trend toward enhanced vascularisation after 4 weeks *in vivo* in the prevascularised microtissues. It would also be interesting to examine vessel infiltration at an earlier time point, prior to the innate upregulation of VEGF by the hypertrophic microtissues.

The concept of using spheroids and microtissues as building blocks to biofabricate larger tissues and organs, although still in its infancy, shows great promise. By employing a modular design when engineering tissues, highly complex systems can be subdivided and individually optimised before being brought together as ideal precursors for organogenesis. To this end, 3D bioprinting offers a means of spatially organising various individual microtissues into a single construct. Successful attempts to structure spheroids using bioprinting has resulted in particularly complex or niche bioprinting approaches (Norotte et al., 2009; Yu et al., 2016; Moldovan et al., 2017; Ayan et al., 2020). Here, we employed a common and simple 3D bioprinting technology, pneumatic extrusion, to deposit our microtissue precursors. The proof of concept provided in this study, successfully deposited microtissues into an anatomically accurate implant, offering an insight into the feasibility of the use of a similar approach for complex, multicellular/tissue systems. Despite this promising outlook, further optimisation is needed to achieve comparable levels of microtissue density within the construct when 3D bioprinting compared to manual deposition or casting.

## Conclusion

To conclude, this study demonstrated a novel method of producing hypertrophic microtissues which undergo mineralisation upon subcutaneous implantation into nude mice. Prevascularising these microtissues with microvessels formed from a co-culture of HUVECs and hBMSCs accelerated mineralisation of the engineered graft in an ectopic model. Prevascularised hypertrophic cartilage spheroids or microtissues represent a promising platform for tissue engineering or 3D bioprinting bone graft substitutes.

## Data Availability Statement

The original contributions presented in the study are included in the article/Supplementary Material, further inquiries can be directed to the corresponding author/s.

## Ethics Statement

The animal study was reviewed and approved by the ethics committee in Trinity College Dublin, and the Health Products Regulatory Authority (HPRA) in Ireland (Approval—AE19136/P087/P069).

## Author Contributions

RB conceived and designed the microwell system. JN carried out *in vitro* studies and analysis, with assistance from RB. JN and RB contributed equally to the *in vivo* study. JN carried out the subsequent data analysis. All authors contributed to the conception and design of the study, contributed to the manuscript and have approved the submitted version.

## Conflict of Interest

The authors declare that the research was conducted in the absence of any commercial or financial relationships that could be construed as a potential conflict of interest.

## Footnotes

1 www.slicer.org

## Supplementary Material

Supplementary Figure 1 Positive mould design and seeding protocol maximises efficiency of microwells. The addition of a chamfer to the top of each well that overlaps effectively collects cells which would otherwise settle on the flat surface above the wells in a “no-chamfer” design. Centrifugation furthers the efficiency of seeding by collecting cells at the bottom of the microwells. (Scale Bar = 500 μm).

Supplementary Figure 2 Cells self-organise into spheroids 15 h after being seeded into the microwells. Still images take of a single microwell containing 2,000 hBMSCs aggregating over the first 21 h of culture in XPAN medium. Each image represents an advancement of 3 h from the last, starting at 0 h. (Scale Bar = 100 μm).

Supplementary Figure 3 Bioprinting proof of principle. **(A)** Design of an anatomically accurate scaffold of a segment of a rat femur. i microCT scan of a rat femur. Ii and iii rat femoral bone segment stl file. iv and v 3D representation of printing design. **(B)** macroscopic image of scaffold prior to implantation **(C)** macroscopic image of scaffold 4-week post-implantation. **(D)** microCT 3D reconstruction of bone volume after 4 weeks *in vivo*. All scale bars = 1 mm.
