# Supplementary material for: Biofabrication of Prevascularised Hypertrophic Cartilage Microtissues for Bone Tissue Engineering
Source: Front Bioeng Biotechnol. 2021 Jun 7;9:661989. doi: 10.3389/fbioe.2021.661989 (PMC8218548; doi:10.3389/fbioe.2021.661989)
Supplement: Supplementary file 1 [file Table_1.DOCX]

%

(RegenHU)

(Generated with BioCad V1.1)

(File: Bone_1_tissue simplified 2)

(http://www.regenhu.com/)

(Comment : Exported from BioCAM)

(Date : 10/24/2019)

(Program-Start)

G90 G94

(PARALLEL PRINTING)

(Beginning [Object 01-Group Bone_1_tissue-Level 0.100-Z = 0.100 Volume Bone_1_tissue])

G0 Z30

M99

M90 P3 D3

M95 P1

G0 X-0.062 Y3.678

G0 Z0.1

M97

F2 G1 X-0.091 Y2.938

X0.705 Y2.938

X0.604 Y2.322

X0.502 Y1.753

X1.485 Y1.517

X1.678 Y1.279

X1.757 Y1.19

X1.896 Y0.802

X1.881 Y0.525

X1.925 Y0.331

X1.876 Y0.09

X1.757 Y-0.352

X1.639 Y-0.72

X1.339 Y-1.074

X0.954 Y-1.371

X0.37 Y-1.712

X0.209 Y-1.774

X-0.001 Y-1.916

X-0.094 Y-1.952

X-0.45 Y-1.712

X-0.472 Y-1.571

X-0.678 Y-0.982

X-0.859 Y-0.403

X-1.027 Y-0.093

X-1.172 Y0.337

X-1.172 Y0.746

X-1.027 Y1.238

X-0.926 Y1.658

X-0.679 Y1.773

X-0.422 Y1.84

X-0.041 Y1.843

X0.263 Y1.754

X0.502 Y1.753

M96

G0 Z0.2

(Ending [Object 01-Group Bone_1_tissue-Level 0.100-Z = 0.100 Volume Bone_1_tissue])

(Beginning [Object 01-Group Bone_1_tissue-Level 0.300-Z = 0.300 Volume Bone_1_tissue])

G0 Z0.42

G0 X0.851 Y1.653

G0 Z0.32

M97

G1 X1.376 Y1.429

X1.701 Y1.157

X1.856 Y0.763

X1.906 Y0.521

X1.906 Y0.201

X1.824 Y-0.234

X1.701 Y-0.423

X1.575 Y-0.757

X1.052 Y-1.232

X0.469 Y-1.671

X0.059 Y-1.857

X-0.064 Y-1.974

X-0.354 Y-1.807

X-0.594 Y-1.232

X-1.119 Y0.031

X-1.21 Y0.555

X-1.112 Y1.128

X-0.918 Y1.618

X-0.745 Y1.741

X-0.322 Y1.745

X-0.074 Y1.769

X0.398 Y1.715

X0.851 Y1.653

M96

G0 Z0.42

(Ending [Object 01-Group Bone_1_tissue-Level 0.300-Z = 0.300 Volume Bone_1_tissue])

(Beginning [Object 01-Group Bone_1_tissue-Level 0.500-Z = 0.500 Volume Bone_1_tissue])

G0 Z0.64

G0 X0.013 Y1.701

G0 Z0.54

M97

G1 X0.398 Y1.652

X0.944 Y1.588

X1.359 Y1.454

X1.727 Y1.073

X1.861 Y0.6

X1.861 Y0.107

X1.754 Y-0.324

X1.615 Y-0.701

X1.2 Y-1.145

X0.759 Y-1.495

X0.523 Y-1.635

X0.398 Y-1.735

X0.026 Y-1.891

X-0.158 Y-2.065

X-0.338 Y-1.922

X-0.587 Y-1.454

X-1.089 Y-0.198

X-1.203 Y0.159

X-1.203 Y0.917

X-1.012 Y1.486

X-0.854 Y1.657

X-0.647 Y1.744

X-0.332 Y1.851

X-0.123 Y1.749

X0.013 Y1.701

M96

G0 Z0.64

(Ending [Object 01-Group Bone_1_tissue-Level 0.500-Z = 0.500 Volume Bone_1_tissue])

(Beginning [Object 01-Group Bone_1_tissue-Level 0.700-Z = 0.700 Volume Bone_1_tissue])

G0 Z0.86

G0 X1.717 Y1.011

G0 Z0.76

M97

G1 X1.837 Y0.726

X1.868 Y0.423

X1.868 Y0.284

X1.766 Y-0.307

X1.613 Y-0.697

X1.395 Y-0.972

X0.87 Y-1.401

X0.374 Y-1.762

X-0.064 Y-1.918

X-0.214 Y-1.972

X-0.353 Y-1.972

X-0.807 Y-1.041

X-1.097 Y-0.186

X-1.259 Y0.083

X-1.259 Y0.642

X-1.097 Y1.143

X-0.93 Y1.563

X-0.445 Y1.769

M96

G0 Z0.86

G0 X-0.447 Y1.765

G0 Z0.76

M97

G1 X-0.214 Y1.812

X-0.119 Y1.671

X0.528 Y1.621

X0.9 Y1.559

X1.401 Y1.339

X1.717 Y1.011

M96

G0 Z0.86

(Ending [Object 01-Group Bone_1_tissue-Level 0.700-Z = 0.700 Volume Bone_1_tissue])

(Beginning [Object 01-Group Bone_1_tissue-Level 0.900-Z = 0.900 Volume Bone_1_tissue])

G0 Z1.08

G0 X-0.287 Y-2.09

G0 Z0.98

M97

G1 X-0.386 Y-2.019

X-0.494 Y-1.858

X-0.784 Y-1.093

X-1.243 Y-0.031

X-1.295 Y0.226

X-1.286 Y0.414

X-1.202 Y0.873

X-1.084 Y1.301

X-1.01 Y1.448

X-0.932 Y1.523

X-0.797 Y1.597

X-0.485 Y1.731

X-0.306 Y1.766

X-0.21 Y1.756

X0.062 Y1.641

X0.07 Y1.656

X0.183 Y1.593

X0.542 Y1.582

X0.924 Y1.503

X1.26 Y1.375

X1.459 Y1.268

X1.621 Y1.118

X1.704 Y0.971

X1.807 Y0.65

X1.852 Y0.271

X1.838 Y0.085

X1.733 Y-0.39

X1.594 Y-0.715

X1.503 Y-0.851

X1.135 Y-1.215

X0.573 Y-1.653

X0.33 Y-1.807

X-0.134 Y-2.051

X-0.288 Y-2.09

M96

G0 Z1.08

(Ending [Object 01-Group Bone_1_tissue-Level 0.900-Z = 0.900 Volume Bone_1_tissue])

(Beginning [Object 01-Group Bone_1_tissue-Level 1.100-Z = 1.100 Volume Bone_1_tissue])

G0 Z1.3

G0 X1.705 Y0.879

G0 Z1.2

M97

G1 X1.799 Y0.469

X1.839 Y0.229

X1.693 Y-0.355

X1.693 Y-0.47

X1.544 Y-0.792

X1.267 Y-1.035

X1.123 Y-1.232

X0.772 Y-1.499

X0.232 Y-1.88

X-0.021 Y-1.999

X-0.208 Y-2.117

X-0.469 Y-1.998

X-0.509 Y-1.921

X-0.914 Y-0.883

X-1.267 Y-0.107

X-1.315 Y0.28

M96

G0 Z1.3

G0 X-1.31 Y0.3

G0 Z1.2

M97

G1 X-1.252 Y0.6

M96

G0 Z1.3

G0 X-1.267 Y0.589

G0 Z1.2

M97

G1 X-1.141 Y1.109

M96

G0 Z1.3

G0 X-1.121 Y1.125

G0 Z1.2

M97

G1 X-0.938 Y1.488

X-0.565 Y1.678

X-0.466 Y1.704

X-0.19 Y1.727

X-0.049 Y1.634

X0.888 Y1.483

M96

G0 Z1.3

G0 X1.467 Y1.211

G0 Z1.2

M97

G1 X1.705 Y0.879

M96

G0 Z1.3

G0 X1.267 Y1.328

G0 Z1.2

M97

G1 X1.467 Y1.211

M96

G0 Z1.3

G0 X0.888 Y1.483

G0 Z1.2

M97

G1 X1.267 Y1.328

M96

G0 Z1.3

(Ending [Object 01-Group Bone_1_tissue-Level 1.100-Z = 1.100 Volume Bone_1_tissue])

(Beginning [Object 01-Group Bone_1_tissue-Level 1.300-Z = 1.300 Volume Bone_1_tissue])

G0 Z1.52

G0 X1.248 Y1.288

G0 Z1.42

M97

G1 X1.461 Y1.162

X1.675 Y0.9

X1.769 Y0.507

X1.769 Y-0.048

X1.675 Y-0.528

X1.543 Y-0.784

X1.354 Y-0.972

X1.026 Y-1.229

X0.841 Y-1.495

X0.568 Y-1.698

X0.287 Y-1.867

X-0.042 Y-2.094

X-0.275 Y-2.18

X-0.468 Y-2.094

X-0.616 Y-1.867

X-0.882 Y-1.048

X-1.304 Y-0.175

X-1.343 Y0.137

X-1.324 Y0.421

X-1.228 Y0.855

X-1.118 Y1.207

X-1.04 Y1.362

X-0.939 Y1.448

X-0.616 Y1.588

X-0.461 Y1.683

X-0.324 Y1.703

X-0.192 Y1.682

X1.244 Y1.3

M96

G0 Z1.52

(Ending [Object 01-Group Bone_1_tissue-Level 1.300-Z = 1.300 Volume Bone_1_tissue])

(Beginning [Object 01-Group Bone_1_tissue-Level 1.500-Z = 1.500 Volume Bone_1_tissue])

G0 Z1.74

G0 X-0.264 Y1.509

G0 Z1.64

M97

G1 X0.126 Y1.512

X0.181 Y1.538

X0.265 Y1.504

X0.581 Y1.468

X0.821 Y1.419

X1.003 Y1.355

X1.452 Y1.13

X1.568 Y1.009

X1.673 Y0.84

X1.757 Y0.542

X1.8 Y0.175

X1.752 Y-0.169

X1.686 Y-0.44

X1.562 Y-0.768

X1.486 Y-0.883

X1.248 Y-1.133

X0.577 Y-1.69

X0.202 Y-1.938

X-0.304 Y-2.203

X-0.37 Y-2.203

X-0.494 Y-2.132

X-0.552 Y-2.04

X-0.882 Y-1.14

X-1.325 Y-0.221

X-1.387 Y0.094

X-1.356 Y0.366

X-1.244 Y0.827

X-1.152 Y1.134

X-1.064 Y1.286

X-0.947 Y1.413

X-0.775 Y1.513

X-0.402 Y1.67

X-0.118 Y1.689

X-0.294 Y1.509

M96

G0 Z1.74

(Ending [Object 01-Group Bone_1_tissue-Level 1.500-Z = 1.500 Volume Bone_1_tissue])

(Beginning [Object 01-Group Bone_1_tissue-Level 1.700-Z = 1.700 Volume Bone_1_tissue])

G0 Z1.96

G0 X1.185 Y1.213

G0 Z1.86

M97

G1 X1.418 Y1.103

X1.546 Y0.985

X1.651 Y0.827

X1.735 Y0.447

X1.735 Y-0.41

M96

G0 Z1.96

G0 X1.714 Y-0.41

G0 Z1.86

M97

G1 X1.396 Y-1.001

X0.861 Y-1.474

X0.429 Y-1.805

X-0.198 Y-2.172

X-0.396 Y-2.244

X-0.546 Y-2.172

X-0.814 Y-1.41

X-1.014 Y-0.867

X-1.37 Y-0.233

X-1.415 Y0.053

X-1.415 Y0.282

X-1.285 Y0.697

X-1.193 Y1.052

X-1.114 Y1.213

X-0.892 Y1.414

X-0.438 Y1.627

X-0.314 Y1.645

X-0.194 Y1.564

X-0.092 Y1.561

X0.06 Y1.489

X0.671 Y1.411

X1.185 Y1.213

M96

G0 Z1.96

(Ending [Object 01-Group Bone_1_tissue-Level 1.700-Z = 1.700 Volume Bone_1_tissue])

(Beginning [Object 01-Group Bone_1_tissue-Level 1.900-Z = 1.900 Volume Bone_1_tissue])

G0 Z2.18

G0 X-0.131 Y1.472

G0 Z2.08

M97

G1 X-0.01 Y1.476

X0.089 Y1.439

X0.511 Y1.4

X0.777 Y1.342

X1.025 Y1.265

X1.422 Y1.057

X1.558 Y0.91

X1.646 Y0.75

X1.733 Y0.435

X1.757 Y0.119

X1.717 Y-0.217

X1.603 Y-0.639

X1.468 Y-0.89

X1.3 Y-1.086

X0.914 Y-1.429

X0.361 Y-1.869

X0.066 Y-2.05

X-0.42 Y-2.301

X-0.449 Y-2.301

X-0.561 Y-2.229

X-0.613 Y-2.147

X-0.743 Y-1.799

X-0.881 Y-1.328

X-1.1 Y-0.857

X-1.382 Y-0.322

X-1.419 Y-0.17

X-1.437 Y0.062

X-1.398 Y0.344

X-1.214 Y1.002

X-1.178 Y1.098

X-1.087 Y1.235

X-0.94 Y1.348

X-0.38 Y1.601

X-0.209 Y1.624

X-0.161 Y1.471

M96

G0 Z2.18

(Ending [Object 01-Group Bone_1_tissue-Level 1.900-Z = 1.900 Volume Bone_1_tissue])

(Beginning [Object 01-Group Bone_1_tissue-Level 2.100-Z = 2.100 Volume Bone_1_tissue])

G0 Z2.4

G0 X-0.476 Y-2.354

G0 Z2.3

M97

G1 X-0.527 Y-2.351

X-0.646 Y-2.179

X-0.914 Y-1.369

X-1.11 Y-0.93

X-1.335 Y-0.535

X-1.406 Y-0.37

X-1.453 Y-0.206

X-1.474 Y-0.018

X-1.42 Y0.312

X-1.236 Y0.952

X-1.156 Y1.116

X-1.095 Y1.196

X-0.948 Y1.309

X-0.422 Y1.571

X-0.279 Y1.611

X-0.243 Y1.528

X-0.141 Y1.457

X-0.026 Y1.462

X0.011 Y1.495

X0.069 Y1.446

X0.298 Y1.385

X0.533 Y1.362

X0.877 Y1.271

X1.297 Y1.081

X1.396 Y1.019

X1.564 Y0.844

X1.636 Y0.703

X1.719 Y0.422

X1.739 Y0.066

X1.698 Y-0.232

X1.592 Y-0.642

X1.497 Y-0.841

X1.272 Y-1.119

X0.541 Y-1.745

X0.306 Y-1.92

X0.018 Y-2.103

X-0.286 Y-2.234

X-0.447 Y-2.348

M96

G0 Z2.4

(Ending [Object 01-Group Bone_1_tissue-Level 2.100-Z = 2.100 Volume Bone_1_tissue])

(Beginning [Object 01-Group Bone_1_tissue-Level 2.300-Z = 2.300 Volume Bone_1_tissue])

G0 Z2.62

G0 X-0.636 Y-2.308

G0 Z2.52

M97

G1 X-0.694 Y-2.207

X-0.803 Y-1.818

X-1.052 Y-1.146

X-1.336 Y-0.636

X-1.484 Y-0.222

X-1.503 Y-0.045

X-1.425 Y0.358

X-1.256 Y0.903

X-1.186 Y1.052

X-1.038 Y1.215

X-0.937 Y1.287

X-0.558 Y1.485

X-0.383 Y1.539

X-0.256 Y1.523

X-0.221 Y1.48

X-0.075 Y1.384

X0.037 Y1.4

X0.116 Y1.37

X0.556 Y1.316

X0.769 Y1.268

X1.091 Y1.143

X1.368 Y0.997

X1.462 Y0.918

X1.614 Y0.689

X1.69 Y0.43

X1.728 Y0.095

X1.688 Y-0.214

X1.585 Y-0.623

X1.469 Y-0.866

X1.31 Y-1.085

X0.917 Y-1.44

X0.234 Y-1.985

X-0.068 Y-2.179

X-0.552 Y-2.374

X-0.636 Y-2.308

M96

G0 Z2.62

(Ending [Object 01-Group Bone_1_tissue-Level 2.300-Z = 2.300 Volume Bone_1_tissue])

(Beginning [Object 01-Group Bone_1_tissue-Level 2.500-Z = 2.500 Volume Bone_1_tissue])

G0 Z2.84

G0 X0.482 Y1.287

G0 Z2.74

M97

G1 X0.747 Y1.233

X1.322 Y0.997

X1.489 Y0.847

X1.596 Y0.666

X1.677 Y0.374

X1.701 Y0.082

X1.665 Y-0.24

X1.588 Y-0.555

X1.467 Y-0.85

X1.354 Y-1.018

X0.939 Y-1.424

X0.166 Y-2.051

X-0.159 Y-2.244

X-0.515 Y-2.397

X-0.574 Y-2.41

X-0.658 Y-2.385

X-0.702 Y-2.308

X-0.864 Y-1.769

X-1.05 Y-1.246

X-1.313 Y-0.765

X-1.516 Y-0.296

X-1.542 Y-0.138

X-1.455 Y0.313

X-1.278 Y0.863

X-1.207 Y1.011

X-1.039 Y1.188

X-0.905 Y1.284

X-0.627 Y1.438

X-0.436 Y1.507

X-0.189 Y1.443

X0.078 Y1.339

X0.453 Y1.293

M96

G0 Z2.84

(Ending [Object 01-Group Bone_1_tissue-Level 2.500-Z = 2.500 Volume Bone_1_tissue])

(Beginning [Object 01-Group Bone_1_tissue-Level 2.700-Z = 2.700 Volume Bone_1_tissue])

G0 Z3.06

G0 X-0.718 Y-2.397

G0 Z2.96

M97

G1 X-1.067 Y-1.314

X-1.37 Y-0.759

X-1.529 Y-0.418

X-1.558 Y-0.264

X-1.558 Y-0.104

X-1.491 Y0.22

X-1.348 Y0.698

X-1.293 Y0.844

X-1.212 Y0.979

X-1.069 Y1.145

X-0.906 Y1.26

X-0.58 Y1.433

X-0.462 Y1.478

X-0.378 Y1.486

X-0.341 Y1.442

X-0.204 Y1.359

X-0.061 Y1.349

X0.117 Y1.286

X0.507 Y1.25

X0.76 Y1.186

X1.344 Y0.934

X1.478 Y0.803

X1.58 Y0.625

X1.66 Y0.328

X1.673 Y0.065

X1.651 Y-0.204

X1.584 Y-0.528

X1.455 Y-0.863

X1.354 Y-1.003

X0.928 Y-1.429

X0.188 Y-2.047

X-0.117 Y-2.245

X-0.656 Y-2.477

X-0.72 Y-2.399

M96

G0 Z3.06

(Ending [Object 01-Group Bone_1_tissue-Level 2.700-Z = 2.700 Volume Bone_1_tissue])

(Beginning [Object 01-Group Bone_1_tissue-Level 2.900-Z = 2.900 Volume Bone_1_tissue])

G0 Z3.28

G0 X-0.756 Y-2.43

G0 Z3.18

M97

G1 X-1.06 Y-1.447

X-1.186 Y-1.169

X-1.454 Y-0.694

X-1.593 Y-0.336

X-1.599 Y-0.239

X-1.517 Y0.195

X-1.329 Y0.784

X-1.269 Y0.89

X-1.086 Y1.109

X-0.945 Y1.219

X-0.607 Y1.392

X-0.447 Y1.437

X-0.206 Y1.343

X-0.106 Y1.324

X0.004 Y1.264

X0.433 Y1.228

X0.689 Y1.167

X1.244 Y0.947

X1.361 Y0.876

X1.486 Y0.745

X1.565 Y0.59

X1.635 Y0.322

X1.647 Y0.022

X1.623 Y-0.274

X1.565 Y-0.553

X1.43 Y-0.885

X1.298 Y-1.049

X0.922 Y-1.438

X0.073 Y-2.148

X-0.192 Y-2.3

X-0.642 Y-2.507

X-0.712 Y-2.505

X-0.756 Y-2.43

M96

G0 Z3.28

(Ending [Object 01-Group Bone_1_tissue-Level 2.900-Z = 2.900 Volume Bone_1_tissue])

(Beginning [Object 01-Group Bone_1_tissue-Level 3.100-Z = 3.100 Volume Bone_1_tissue])

G0 Z3.5

G0 X-0.762 Y-2.523

G0 Z3.4

M97

G1 X-0.802 Y-2.448

X-1.083 Y-1.507

X-1.19 Y-1.264

X-1.476 Y-0.756

X-1.574 Y-0.527

X-1.624 Y-0.343

X-1.622 Y-0.201

X-1.563 Y0.112

X-1.392 Y0.656

X-1.297 Y0.843

X-1.153 Y1.025

X-0.958 Y1.188

X-0.691 Y1.322

X-0.52 Y1.386

X-0.338 Y1.389

X-0.219 Y1.349

X-0.198 Y1.286

X-0.096 Y1.248

X0.06 Y1.254

X0.521 Y1.172

X0.796 Y1.09

X1.19 Y0.929

X1.39 Y0.803

X1.494 Y0.675

X1.574 Y0.44

X1.623 Y0.126

X1.613 Y-0.175

X1.547 Y-0.546

X1.423 Y-0.871

X1.337 Y-1.015

X0.875 Y-1.48

X0.023 Y-2.2

X-0.254 Y-2.356

X-0.711 Y-2.575

X-0.762 Y-2.523

M96

G0 Z3.5

(Ending [Object 01-Group Bone_1_tissue-Level 3.100-Z = 3.100 Volume Bone_1_tissue])

(Beginning [Object 01-Group Bone_1_tissue-Level 3.300-Z = 3.300 Volume Bone_1_tissue])

G0 Z3.72

G0 X-0.817 Y-2.552

G0 Z3.62

M97

G1 X-1.125 Y-1.505

X-1.63 Y-0.532

X-1.654 Y-0.283

X-1.636 Y-0.086

X-1.396 Y0.661

X-1.33 Y0.793

X-1.192 Y0.957

X-0.959 Y1.161

X-0.515 Y1.379

X-0.214 Y1.299

X-0.108 Y1.232

X0.397 Y1.167

X0.672 Y1.095

X1.213 Y0.876

X1.384 Y0.761

X1.497 Y0.587

X1.589 Y0.246

X1.603 Y-0.038

X1.525 Y-0.535

X1.377 Y-0.923

X1.242 Y-1.113

X1.055 Y-1.309

X0.606 Y-1.719

X-0.022 Y-2.249

X-0.503 Y-2.512

X-0.773 Y-2.627

X-0.815 Y-2.553

M96

G0 Z3.72

(Ending [Object 01-Group Bone_1_tissue-Level 3.300-Z = 3.300 Volume Bone_1_tissue])

(Beginning [Object 01-Group Bone_1_tissue-Level 3.500-Z = 3.500 Volume Bone_1_tissue])

G0 Z3.94

G0 X-0.75 Y-2.651

G0 Z3.84

M97

G1 X-0.824 Y-2.669

X-0.856 Y-2.591

X-1.18 Y-1.466

X-1.618 Y-0.694

X-1.664 Y-0.55

X-1.684 Y-0.308

X-1.651 Y-0.045

X-1.359 Y0.762

X-1.215 Y0.946

X-1.019 Y1.107

X-0.638 Y1.322

X-0.532 Y1.347

X-0.47 Y1.32

X-0.263 Y1.286

X-0.199 Y1.174

X-0.032 Y1.215

X0.052 Y1.181

X0.428 Y1.125

X0.678 Y1.064

X0.911 Y0.978

X1.251 Y0.81

X1.344 Y0.741

X1.453 Y0.603

X1.509 Y0.467

X1.564 Y0.205

X1.561 Y-0.106

X1.5 Y-0.559

X1.367 Y-0.932

X1.238 Y-1.103

X0.94 Y-1.417

X0.035 Y-2.224

X-0.407 Y-2.494

X-0.749 Y-2.65

M96

G0 Z3.94

(Ending [Object 01-Group Bone_1_tissue-Level 3.500-Z = 3.500 Volume Bone_1_tissue])

(Beginning [Object 01-Group Bone_1_tissue-Level 3.700-Z = 3.700 Volume Bone_1_tissue])

G0 Z4.16

G0 X-0.282 Y1.194

G0 Z4.06

M97

G1 X0.007 Y1.144

X0.304 Y1.144

X0.607 Y1.025

X1.211 Y0.777

X1.42 Y0.558

X1.531 Y0.227

X1.531 Y-0.122

X1.544 Y-0.3

X1.508 Y-0.536

X1.437 Y-0.746

X1.274 Y-1.028

X1.066 Y-1.284

X0.744 Y-1.625

X0.108 Y-2.139

X-0.289 Y-2.452

X-0.752 Y-2.695

X-0.87 Y-2.676

X-0.87 Y-2.452

X-1.166 Y-1.625

X-1.251 Y-1.445

X-1.673 Y-0.621

X-1.699 Y-0.285

X-1.655 Y0.03

X-1.409 Y0.558

X-1.251 Y0.89

X-0.935 Y1.132

X-0.626 Y1.29

X-0.472 Y1.294

X-0.399 Y1.319

X-0.282 Y1.194

M96

G0 Z4.16

M98

(Ending [Object 01-Group Bone_1_tissue-Level 3.700-Z = 3.700 Volume Bone_1_tissue])

(Program-End)

G0 Z30

G0 X0 Y0

M2
