# Supplementary figures and images for: Biofabrication of Prevascularised Hypertrophic Cartilage Microtissues for Bone Tissue Engineering
Source: Front Bioeng Biotechnol. 2021 Jun 7;9:661989. doi: 10.3389/fbioe.2021.661989 (PMC8218548; doi:10.3389/fbioe.2021.661989)

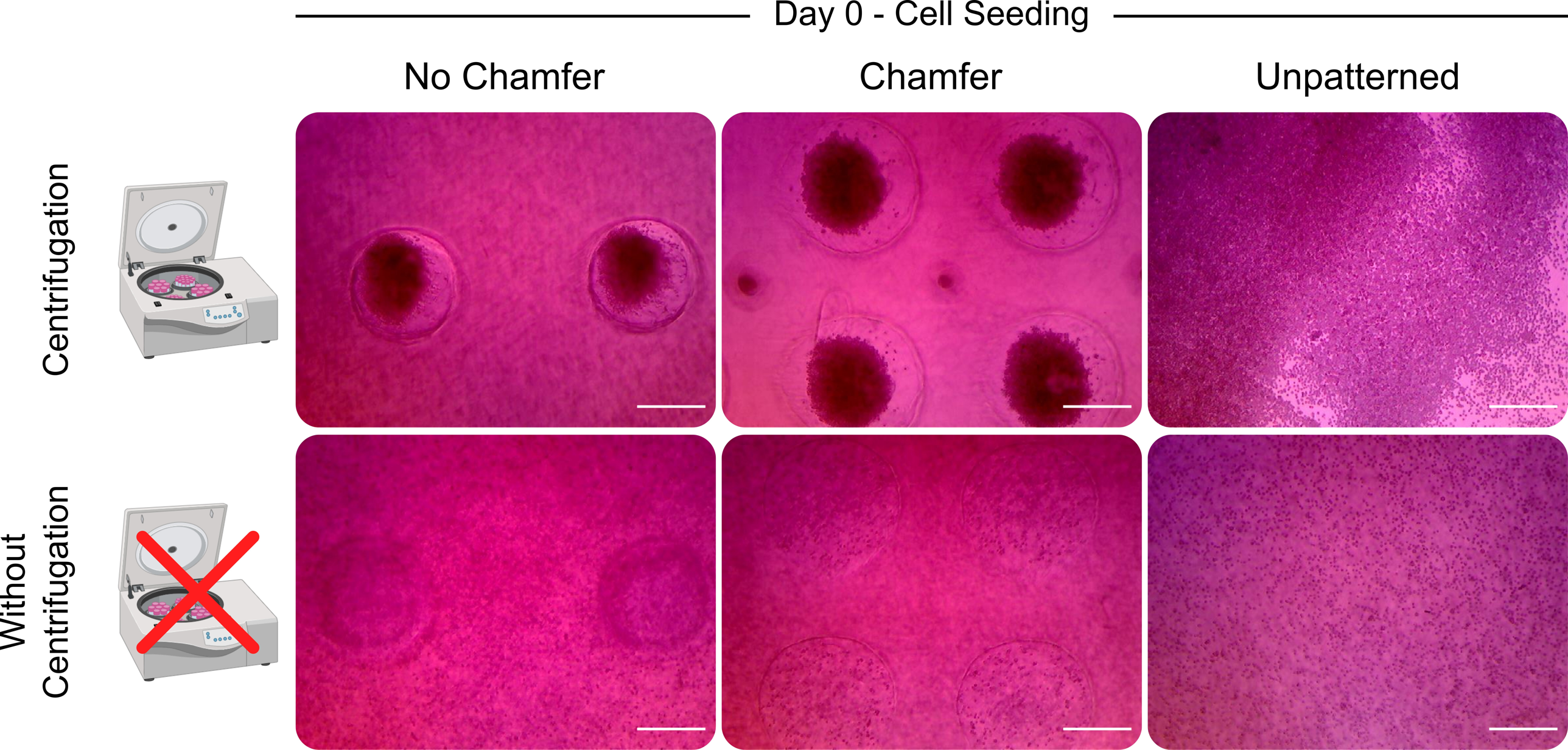

Supplement: Supplementary Figure 1 — Positive mould design and seeding protocol maximises efficiency of microwells. The addition of a chamfer to the top of each well that overlaps effectively collects cells which would otherwise settle on the flat surface above the wells in a “no-chamfer” design. Centrifugation furthers the efficiency of seeding by collecting cells at the bottom of the microwells. (Scale Bar = 500 μm). [file Image_1.JPEG]

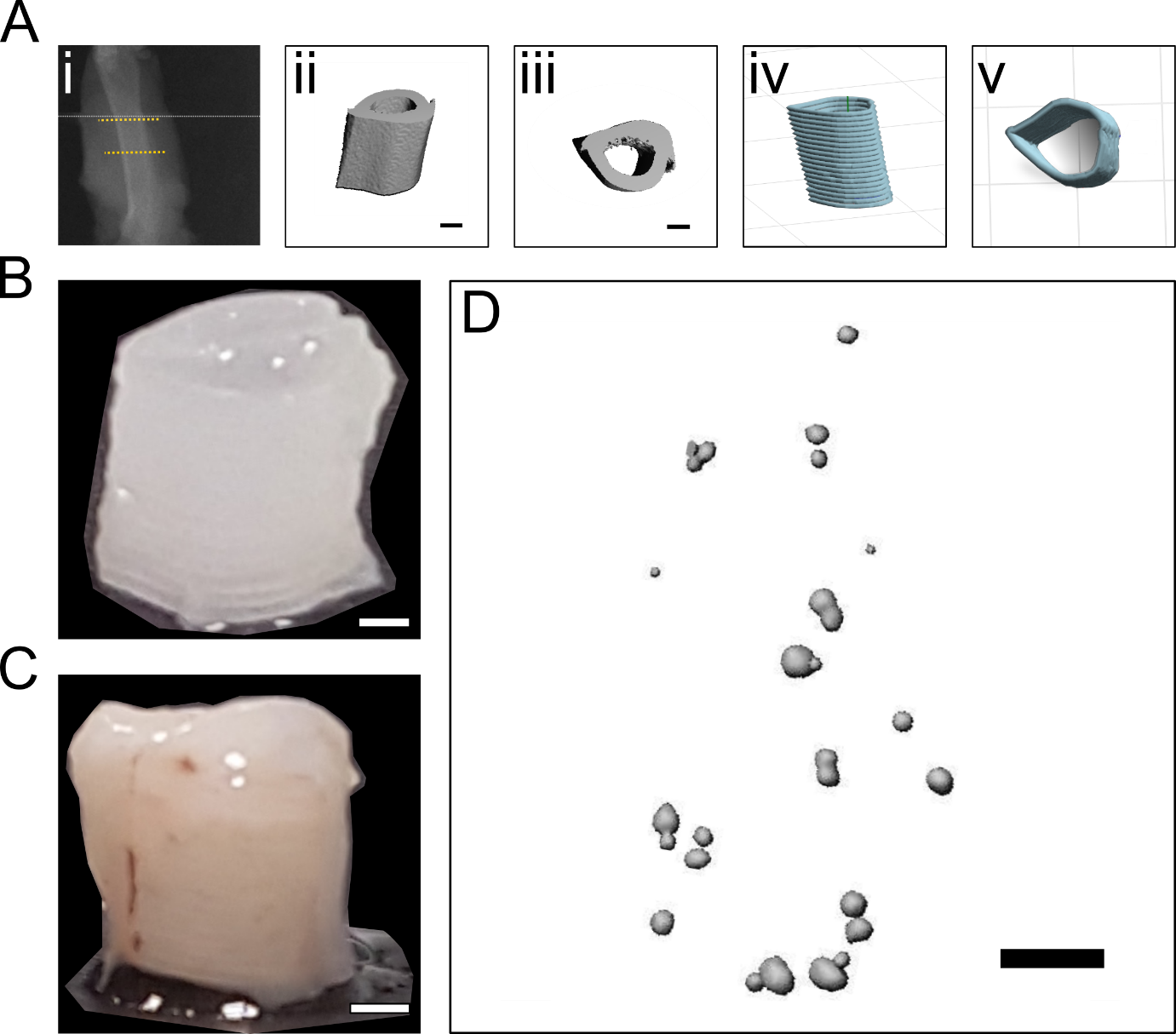

Supplement: Supplementary Figure 3 — Bioprinting proof of principle. (A) Design of an anatomically accurate scaffold of a segment of a rat femur. i microCT scan of a rat femur. Ii and iii rat femoral bone segment stl file. iv and v 3D representation of printing design. (B) macroscopic image of scaffold prior to implantation (C) macroscopic image of scaffold 4-week post-implantation. (D) microCT 3D reconstruction of bone volume after 4 weeks in vivo. All scale bars = 1 mm. [file Image_3.TIF]
